# High-Update-Rate (25 kHz) Laser Ranging with Random Noise Modulation for Fast and Precise Absolute Distance Measurement

**DOI:** 10.3390/s25226985

**Published:** 2025-11-15

**Authors:** Yongchao Chen, Tianci Liu, Shenggang Liu, Longhuang Tang, Heli Ma, Tianjiong Tao, Xing Jia, Jian Wu, Chengjun Li, Xiang Wang, Zongchen Wang, Weilu Chen, Xirui Zhang, Entao Li, Jia Shi, Jidong Weng

**Affiliations:** 1 National Key Laboratory of Shock Wave and Detonation Physics, Institute of Fluid Physics, China Academy of Engineering Physics, Mianyang 621000, China; ycchen16@fudan.edu.cn (Y.C.); 2331080969@tiangong.edu.cn (T.L.); liushenggangpla@126.com (S.L.); macos12@126.com (H.M.); zjuttj@163.com (T.T.); jiaxing@caep.cn (X.J.); ceuwj@zju.edu.cn (J.W.); lstrus@126.com (C.L.); xiangwang102@126.com (X.W.); 2331080997@tiangong.edu.cn (Z.W.); chenwlu@mail2.sysu.edu.cn (W.C.); yqq20211107@163.com (X.Z.); 2 Tianjin Key Laboratory of Optoelectronic Detection Technology and System, School of Electronic and Information Engineering, Tiangong University, Tianjin 300387, China; lientao512@163.com (E.L.); shijia@tiangong.edu.cn (J.S.); 3 Taihang Laboratory, Chengdu 610200, China

**Keywords:** random noise, autocorrelation, fast measurement, absolute distance measurement

## Abstract

This paper proposes a technique for precise distance measurement under laser random noise using autocorrelation demodulation. We conducted measurements within a 2 m range using a commercial digitizer with a sampling rate of 40 GS/s. The single signal acquisition time is 40 μs, and the measurement results achieved an accuracy of 30 μm and a standard deviation (SD) of 8 μm. This method can also improve measurement accuracy by increasing the sampling rate and expanding the measurement range by extending the duration of a single sampling.

## 1. Introduction

In the field of precision engineering in manufacturing, such as spacecraft production and large-scale structural health monitoring, high-precision distance measurement technology has always been a core aspect ensuring product quality and safety [1,2,3]. Over the past few decades, optical measurement techniques have continuously evolved, from improvements in traditional methods like Fringe Projection Profilometry (FPP) [4] to the introduction of vortex light [5] and deep learning [6]. However, existing technologies still face trade-offs between measurement range, accuracy, efficiency, and operational complexity, and a universal solution has yet to be established [7,8].

Due to its micron-level accuracy and anti-electromagnetic interference characteristics, optical interferometry has become the mainstream solution for precision measurement [9,10,11]. However, its strict requirements for the manufacturing tolerances of optical components and environmental stability have significantly increased the difficulty of engineering applications. The optical frequency domain interferometry (OFDI) based on a spectrometer simplifies the system structure. However, due to the response speed limitation of the spectrometer, its theoretical maximum measurement distance is only 0.24 m [12,13], making it difficult to cover the common requirements in industrial scenarios ranging from the meter level to the hundred-meter level. Frequency scanning interferometry can achieve high precision in the meter range [14,15,16,17]. However, the contradiction between its measurement range and dynamic response speed restricts its practicality under complex working conditions. In recent years, the femtosecond optical frequency comb combined with the dual-comb improvement scheme has improved the absolute ranging accuracy to the nanometer level, and its feasibility has been verified in the meter range [18,19,20]. However, the measurable range of the direct dual-comb is limited by its repetition frequency. Usually, when the repetition frequency is 100 MHz, the accurately measurable distance is only about 1.5 m, which restricts the measurement applications for long distances [21,22,23]. Although the unambiguous ranging range can be extended to the kilometer level by changing the comb repetition frequency for two independent range measurements, this scheme undermines the real-time performance of dual-comb ranging and cannot perform real-time tracking and measurement of the absolute distance of moving objects. Therefore, the development of a ranging method that combines a large range, high accuracy, fast response and strong environmental adaptability has become an urgent need to promote the technological upgrading of high-end manufacturing [24,25].

In recent years, lidar technologies based on related methods, such as pseudo-random binary sequence (PRBS) lidar and optical chaos lidar, have been proven to achieve centimeter-level ranging accuracy. However, PRBS lidar uses predefined, periodically repeated binary sequences, whose “randomness” is artificially designed and periodic, so its maximum unambiguous measurement range is limited by the sequence length [26]. Optical chaos lidar usually utilizes chaotic carriers generated by the internal dynamics of lasers [27,28]. Nevertheless, compared with traditional pulse lidar, chaos lidar has a low pulse peak power, which limits the signal-to-noise ratio and thus the ranging distance.

In contrast, the randomness of broadband amplified spontaneous emission (ASE) light sources originates from the quantum process of spontaneous emission and is not sensitive to drifts in laser operating parameters or external optical feedback [29]. This kind of signal has an extremely wide continuous frequency spectrum and is inherently aperiodic. This inherently true random statistical characteristic fundamentally avoids the distance ambiguity problem inherent in periodic modulation signals, and in theory, can achieve an infinitely large unambiguous measurement range.

This paper proposes an absolute distance measurement method that generates random noise through ASE and performs demodulation via autocorrelation. Through theoretical modeling and numerical simulations, we demonstrate that when two coherent laser beams (with a fixed optical path difference) are modulated by noise, autocorrelation can extract delay information-the absolute distance can be derived from the autocorrelation peak of their combined signal. The effectiveness of this method was verified using an experimental system built with commercial instruments. We further conducted comparative tests using digitizers with different sampling rates and storage depths, and systematically analyzed the impact of target distance and beam splitting ratio on measurement accuracy. Based on the optimal experimental conditions obtained from these comparative experiments, we performed 100 repeated measurements within a 2 m measurement range. The measurement results show an accuracy of 30 μm with a standard deviation of 8 μm. In addition, each measurement only takes 40 microseconds, achieving high precision and rapid measurement. Compared with the prior art presented in Table 1, this technology is expected to be applicable in fields such as high-frequency vibration monitoring and explosion shock wave measurement.

## 2. Theoretical Analysis

### 2.1. Ranging Principle of Ultra-Fast Random Noise

**Figure 1:**
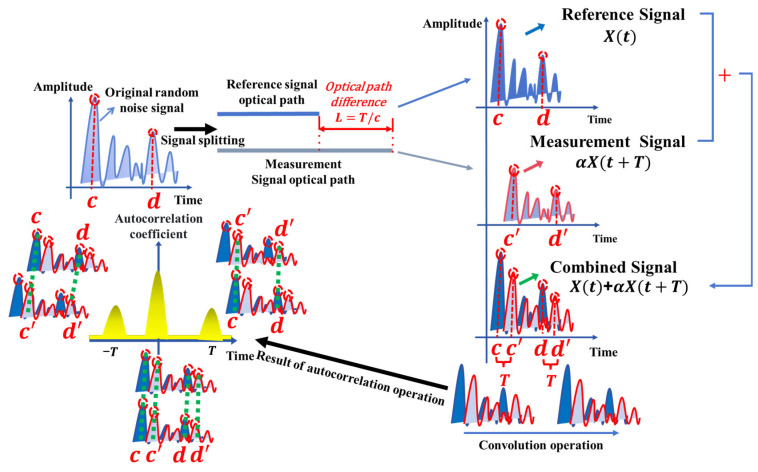
Schematic diagram of autocorrelation system.

Based on the basic principle of time-of-flight measurement, this paper proposes a coaxial optical path ranging method using a random noise light source. The core of this method is to accurately extract the time delay between the reference signal and the measurement signal through autocorrelation processing technology, thereby achieving high-precision absolute distance measurement. This scheme adopts a unique coaxial optical path structure: first, a broadband random noise light source is used to generate an optical signal with excellent autocorrelation characteristics; then, the light source is split into two completely homologous signals by a beam splitter, where the reference signal x(t) is used to establish a time benchmark, and the measurement signal x(t+T) produces a time delay T after being reflected by the measured target; finally, these two signals are combined to form a mixed signal containing time delay information. The combined signal enters the photoelectric converter together. This coaxial structure ensures the spatial consistency and time synchronization of the signals, significantly reducing the inherent alignment errors and environmental interference in traditional dual optical path systems, and thus providing a reliable signal foundation for subsequent time delay extraction. The principle of this method is shown in Figure 1.

#### 2.1.1. Mathematical Modeling of Signals

Based on the aforementioned measurement principles, we proceed to establish a theoretical model of the proposed method.

For the processing of combined signals, we adopted an autocorrelation demodulation method. With this method, autocorrelation calculation is performed on the combined signal containing the reference signal and the measurement signal in the continuous time domain. Its expression is as follows:

(1) $$\int_{-\infty }^{+\infty }\left[x(t)+\alpha x(t+T)][x(t+\tau )+\alpha x(t+T+\tau )\right]\;dt$$

Here, x(t) describes the reference optical signal in the time domain, serving as the original waveform. The coefficient α quantifies the amplitude reduction experienced by the measurement signal relative to the reference. The crucial parameter  T represents the optical time delay incurred by the measurement signal as it travels the additional round-trip path to the target. Finally, τ is a variable time shift introduced in the autocorrelation process. By identifying the peaks in the correlation function, the delay T is determined through convolution operations.

Expanding the calculation based on the definition of the autocorrelation function yields:

(2) $$\left(1+\alpha^{2})R_{xx}(\tau )+\alpha R_{xx}(\tau +T)+\alpha R_{xx}(\tau -T\right)$$

where $R_{xx}(\tau )=\int_{-\infty }^{+\infty }x(t)x(t+\tau )\;dt$ represents the autocorrelation function of the reference optical signal. In this result, the term $\left(1+\alpha^{2})R_{xx}(\tau \right)$ corresponds to the autocorrelation components of the reference and signal lights, centered at τ=0, while the terms $\alpha R_{xx}(\tau +T)$ and $\;\alpha R_{xx}(\tau -T)$ represent cross-correlation components, which produce symmetric peaks at τ=−T and τ=T, respectively.

In actual sampling, the result of discretized autocorrelation is:

(3) $$\left(1+\alpha^{2})R_{xx}[m]+\alpha R_{xx}[m+k]+\alpha R_{xx}[m-k\right]$$

k: the discrete delay quantity in units of sampling points. This parameter is equivalent to the discrete form of the continuous-time delay T, and the two are related through the sampling rate $f_{s}$*:*$k=T\cdot f_{s}$. α: the amplitude attenuation coefficient, whose meaning and function are exactly the same as those of the coefficient in the continuous model. m is the discrete time shift (m=…,−2,−1,0,1,2,…). Its function is the same as that of the continuous variable τ. $R_{xx}[m]$ is the discrete autocorrelation function of the reference optical sequence x[t].

In the discrete autocorrelation sequence ${\;R}_{ss}[m]$, information peaks appear at  m=±k. Once k is precisely determined via peak detection algorithms, the physical optical path difference L can be retrieved through the following relations:

(4) $$L=\frac{T\cdot c}{2}=\frac{k\cdot c}{{2f}_{s}}$$

where c is the speed of light, and the factor of 2 originates from the round-trip propagation of light in the measurement path.

This formula clearly demonstrates that the sampling rate $f_{s}$ determines the distance resolution of the system. The minimum resolvable distance variation is given by $\;\delta L=\frac{c}{{2f}_{s}}$. The discrete delay quantity k thus serves as the crucial bridge connecting digital signal processing to the physical world.

In the discrete sampling architecture of this system, the determination of the optical path difference L ultimately relies on the accurate extraction of the peak time k of the autocorrelation function.

From the perspective of algorithm implementation, to unambiguously identify the peak point *k* representing the optical path difference, it is necessary to ensure that the number of data points captured in a single sampling instance exceeds k. In the time domain, this condition is equivalent to requiring that the signal delay time T must be less than the single sampling duration $T_{\mathrm{acq}}=\frac{N}{f_{s}}\;$. (N: The total number of sampling points = storage depth).

Converting this temporal relationship into an optical path difference, we obtain the fundamental constraint for the system’s measurement range $L_{\max}$:

(5) $$L_{\max}<\frac{c\cdot T_{\mathrm{acq}}}{2}=\frac{c\cdot N}{2f_{s}}$$

Equation (5) shows that the maximum measurable optical path difference in the system is determined by the single sampling duration $T_{\mathrm{acq}}$. This equation unifies the intrinsic correlation among hardware storage depth, sampling rate, and the algorithm’s peak extraction capability, collectively defining the absolute upper limit of the system’s measurable range.

In discrete signal processing, the concept of signal overlap ratio becomes particularly important. The overlap ratio η can be defined as:

(6) $$\eta =\frac{N-|k|}{N}=1-\frac{|k|}{N}$$

This overlap ratio directly influences the signal-to-noise ratio (SNR) of the autocorrelation result. A higher η means a greater number of data points participate in the effective averaging process, thereby enhancing the SNR of the correlation peak and consequently improving the measurement precision. This quantitative relationship explains why, when measuring larger distances (corresponding to a larger k), it is necessary to increase the storage depth N (i.e., extend the single sampling duration $T_{\mathrm{acq}}$) to maintain a high overlap ratio and ensure sufficient measurement accuracy.

#### 2.1.2. Signal-to-Noise Ratio Analysis

In our measurement, we calculate the distance by identifying the positions of these two cross-term peaks. Therefore, the amplitude (i.e., height) of the cross-term peaks is crucial. The higher the amplitude, the sharper the peak, the easier it is to identify and precisely locate, and the stronger the noise resistance, meaning the higher the signal-to-noise ratio.

We begin from the derivation of Formula (2): $\left(1+\alpha^{2})R_{xx}(\tau )+\alpha R_{xx}(\tau +T)+\alpha R_{xx}(\tau -T\right)$. Here, $\left(1+\alpha^{2})R_{xx}(\tau \right)$ is the auto-correlation term. Its peak is located at τ=0, representing the correlation of the signal with itself; $\alpha R_{xx}(\tau +T)$ and $\alpha R_{xx}(\tau -T)$ are cross terms. The peaks of these two terms are located at τ=−T and  τ=T respectively, and they directly contain the required delay information T.

It can be seen that the amplitude of the cross-term peaks is proportional to its coefficient α. Therefore, if the measurement signal is too weak (α is very small), although the auto-correlation term will also become smaller, the absolute value of the cross-term will also be very small. If the measurement signal is too strong (α is very large), the auto-correlation term will become very prominent and may submerge the relatively smaller cross-term.

We find this optimal balance point by calculating the ratio of the cross-term peak amplitude to the auto-correlation term peak amplitude, where cross-term peak amplitude: proportional to $\;\alpha R_{xx}(0)$ (because when τ=T, $R_{xx}(0)$ is the maximum value of the auto-correlation function). Auto-correlation term peak amplitude: located at  τ=0, its amplitude is $\left(1+\alpha^{2})R_{xx}(0\right)$.

A main-to-side-lobe ratio coefficient is constructed, which describes the relative height of the cross-term (side lobe) relative to the auto-correlation term (main lobe):

(7) $$\frac{\mathrm{Cross}\text{-}\mathrm{term}\;\mathrm{Peak}\;\mathrm{Amplitude}}{\mathrm{Auto}\text{-}\mathrm{correlation}\;\mathrm{Term}\;\mathrm{Peak}\;\mathrm{Amplitude}}=\frac{\alpha \cdot R_{xx}(0)}{\left(1+\alpha^{2})\cdot R_{xx}(0\right)}=\frac{\alpha }{1+\alpha^{2}}$$

Using the fundamental inequality in mathematics, for  α>0: $\alpha +\frac{1}{\alpha }\geq 2$. The equality holds if and only if  α=1.

Therefore:

(8) $$\frac{\alpha }{1+\alpha^{2}}=\frac{1}{\alpha +\frac{1}{\alpha }}\leq \frac{1}{2}$$

Similarly, if and only when α=1, this ratio achieves its maximum value of 1/2. Thus, when α=1, the relative height of the cross-term peak relative to the auto-correlation term main peak reaches its maximum, which is 1/2.

### 2.2. Simulation Analysis

This study developed a comprehensive numerical simulation experimental system to validate the ranging performance of the autocorrelation demodulation algorithm in laser random noise modulation environments. The system employs random Gaussian white noise as the reference optical path signal $s_{\mathrm{ref}}\left(t\right)$, and establishes the measurement optical path signal $s_{\mathrm{sig}}\left(t\right)=s_{\mathrm{ref}}\left(t-T\right)$ by introducing a precisely controllable time delay T. The two signals are coupled to form a combined signal $s_{\mathrm{mix}}\left(t\right)=s_{\mathrm{ref}}\left(t\right)+\alpha s_{\mathrm{ref}}\left(t-T\right)$, where α characterizes the optical path attenuation coefficient. Discrete autocorrelation operation is performed on the combined signal to obtain $R_{ss}(m)=\frac{1}{N}\sum_{k=0}^{N-1}s_{\mathrm{mix}}(n)s_{\mathrm{mix}}(n-m)$, where N represents the total number of sampling points and m is the delay variable.

**Figure 2:**
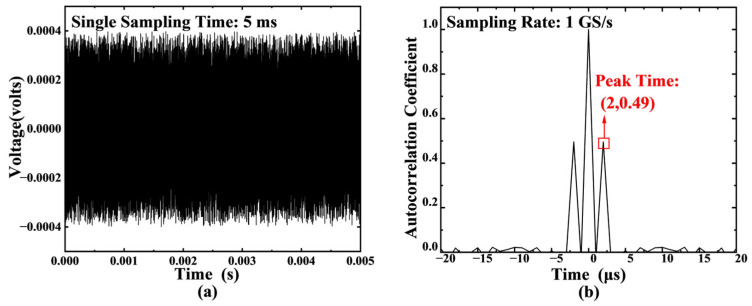
(**a**) Simulated time-domain waveform of the original random noise signal. (**b**) Autocorrelation result of the combined signal, showing the characteristic central peak and symmetric side peaks. Simulation parameters: time delay T = 2 µs, sampling rate = 1 GS/s, acquisition duration = 5 ms.

Under the condition of a set delay time T = 2 μs with a sampling rate of 1 GS/s, a combined signal lasting 5 ms (corresponding to the single sampling duration) was generated for simulation verification. Figure 2a clearly demonstrates the time-domain characteristics of the original random noise signal, while Figure 2b shows distinct conjugate symmetric peaks in the autocorrelation function curve of the combined signal. Through precise extraction, the delay amounts corresponding to the symmetric peaks were determined to be −2 μs and 2 μs, respectively, which completely match the preset parameters, thereby fully validating the theoretical feasibility of the method.

**Figure 3:**
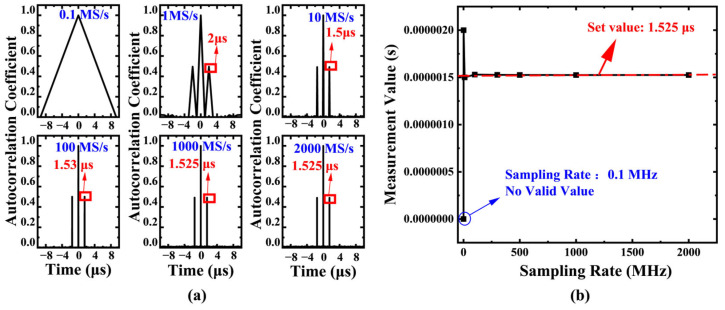
(**a**) Influence of sampling rate on the position of the autocorrelation peak (Fixed parameters: T = 1.525 µs, duration = 10 µs). (**b**) Measure the relationship between the absolute deviation and relative error of time delay and the sampling rate. As the sampling rate increases from 0.1 MS/s to 2000 MS/s, the absolute deviation between the measured delay value and the theoretical delay value decreases monotonically, and the maximum relative error drops from 31.15% to 0%.

The controlled variable method was systematically employed to investigate the influence of sampling rate on measurement accuracy. Under conditions where other parameters remained constant, six sampling rate levels ranging from 0.1 to 2000 MS/s were established, with a single sampling duration set to 10 μs and a fixed delay of 1.525 μs. The simulation results in Figure 3 demonstrate that as the sampling rate increases from 0.1 MS/s to 2000 MS/s, the absolute deviation between the measured delay values and theoretical values shows a monotonically decreasing trend, with the maximum relative error significantly reduced from 31.15% to 0%, confirming the decisive impact of sampling rate on measurement accuracy.

**Figure 4:**
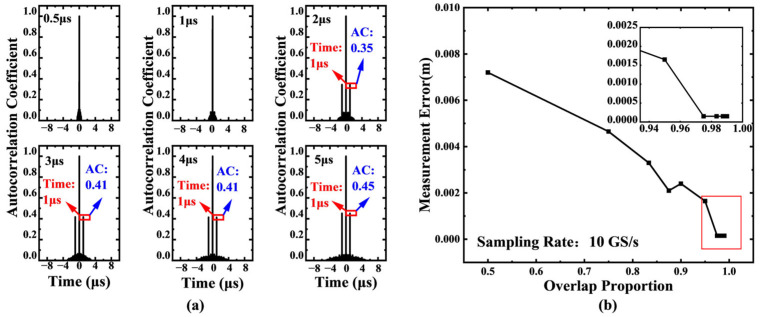
(**a**) System performance under different storage depths with fixed sampling rate (100 MS/s). When the storage depth (acquisition duration) is less than the delay (1 μs), the system fails to detect the delay peak. Effective detection is achieved when the storage depth increases to 2–5 μs, validating the fundamental constraint $L<\frac{c\cdot N}{{2f}_{s}}$ for unambiguous ranging. (**b**) Influence of signal overlap ratio $\eta =1-\frac{|k|}{N}$ on measurement error. As the overlap ratio increases, the measurement error decreases rapidly before stabilizing, confirming that a higher overlap ratio enhances the signal-to-noise ratio of the autocorrelation peak. Simulation parameters: delay = 10 ns, sampling rate = 10 GS/s.

In the study of storage depth effects, with the sampling rate fixed at 100 MS/s, six storage depth levels ranging from 0.5 μs to 5 μs were tested. Figure 4a clearly illustrates the correlation between storage depth and delay detection capability: when the storage depth is less than the preset delay value (1 μs), the system completely fails to detect the delay peak; when the storage depth increases to 2–5 μs, the system can effectively capture the characteristic peak signal. This phenomenon validates the fundamental constraint that the signal acquisition length *L* must satisfy $L<\frac{c\cdot N}{{2f}_{s}}$.

The research further reveals the crucial role of signal overlap ratio. In actual sampling processes, the reference signal length and delayed signal length determine the effective overlap ratio $\eta =1-\frac{|k|}{N}$. Simulation data indicate that improving the overlap ratio directly enhances the signal-to-noise ratio characteristics of the autocorrelation curve. Figure 4b (parameters: delay = 10 ns, sampling rate = 10 GS/s) specifically demonstrates that as the overlap ratio increases, the measurement error shows a pattern of initial rapid decrease followed by gradual stabilization. This characteristic provides important guidance for system parameter optimization.

## 3. Experimental Setup

### 3.1. Time Delay Line Simulation Verification Experiment

To validate the method’s practical feasibility, a proof-of-concept experiment was conducted for the laser-random-noise-based autocorrelation ranging system under laboratory conditions. The experiment systematically evaluated how sampling rate, storage depth, and beam splitter ratio affect measurement accuracy.

**Figure 5:**
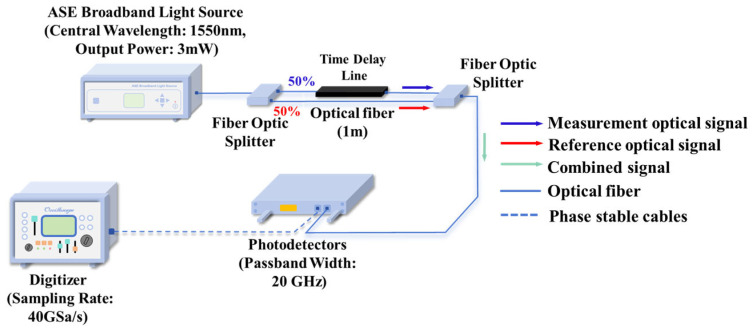
Experimental setup for autocorrelation-based distance measurement using a broadband ASE source (1550 nm). The system employs a 50:50 optical splitter to create reference and measurement paths, with a tunable delay line for path control. Combined signals are detected by a 20 GHz photodetector and acquired by a digitizer. This configuration successfully validates the autocorrelation principle, demonstrating that precise ranging can be achieved through intensity correlation of random noise signals.

The autocorrelation ranging verification system constructed in this study employs a broadband amplified spontaneous emission (ASE) light source (ZIGUAN Inc. ZG-CL10-9-0-M, Spectral Range: 1530–1565 nm, Central Wavelength: 1550 nm, Output Power: 3 mW) as the random signal source [35], establishing a dual optical path structure for reference and measurement through a 50:50 beam splitter. The reference optical path utilizes a 1 m single-mode optical fiber as the baseline path, while the measurement optical path achieves precise optical path control via a tunable delay line. The two optical signals are combined by a coupler (50%:50%) and then converted into time-domain electrical signals by a high-speed photodetector (CONQUER Inc. KGPT, passband width 20 GHz, response wavelength: 1200–1650 nm), with final data acquisition completed by a digitizer. The system architecture is shown in Figure 5.

**Figure 6:**
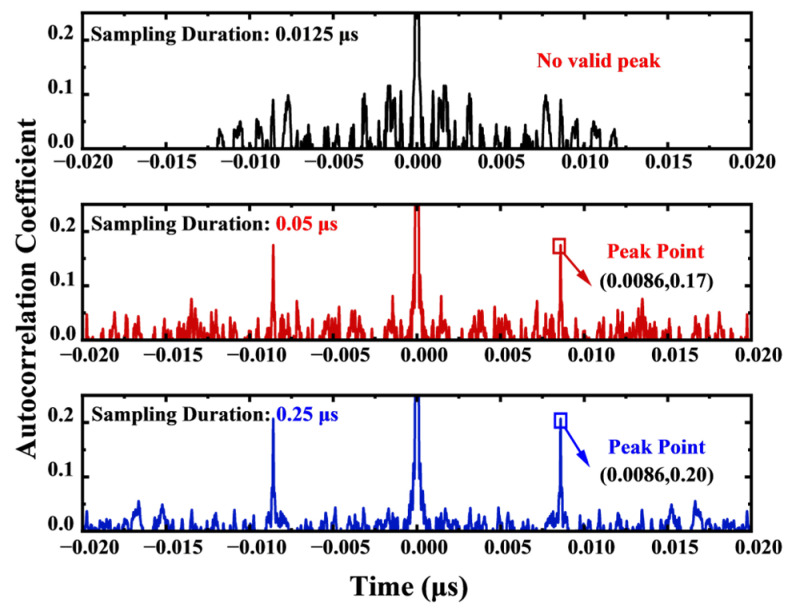
Comparison of measurement results under different sampling durations (12.5 ns, 50 ns, and 250 ns) at a fixed distance of 1.3 m (theoretical delay: 8.6 ns). The results clearly demonstrate that increasing the sampling duration, which expands the signal overlap area, significantly enhances the signal-to-noise ratio, as evidenced by the emergence and growth of the delay peak.

In the storage depth comparison experiment, under the condition of a set test distance of 1.3 m (corresponding to a theoretical delay of 8.6 ns), we systematically compared the measurement effects of three different sampling durations: 12.5 ns, 50 ns, and 250 ns. According to the experimental results shown in Figure 6, the increase in sampling duration significantly improved signal quality characteristics. When the sampling duration was extended from 12.5 ns to 250 ns, the delay point peak increased from an initially undetectable state to a significant level of 0.20. This phenomenon fully confirms that ensuring sufficient signal overlap area plays a crucial role in enhancing the system’s signal-to-noise ratio.

In the study of sampling rate effects, while maintaining other experimental parameters constant, we conducted systematic verification using four different sampling rate levels: 1.25 GS/s, 10 GS/s, 20 GS/s, and 40 GS/s. The experimental data in Table 2 clearly demonstrate that as the sampling rate increased from 10 GS/s to 40 GS/s, the standard deviation of the measurement system significantly decreased from 25,442 μm to 96 μm, representing a measurement accuracy improvement of 99.6%. This result strongly validates the important role of high sampling rates in enhancing the system’s temporal resolution.

**Figure 7:**
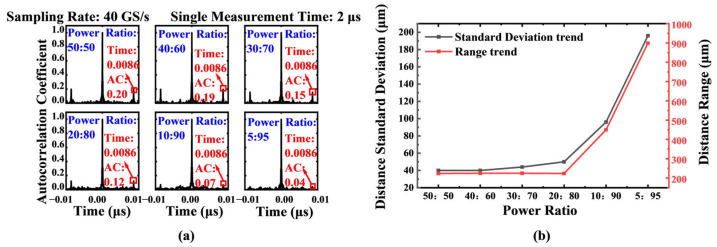
(**a**) Influence of different splitting ratios on the autocorrelation peak.Measured ranging accuracy under different reference-to-signal optical power ratios (from 50:50 to 5:95).; (**b**) Variation trend diagram of standard deviation and range of measurement results under different splitting ratios. The results demonstrate that a power ratio close to 1:1 yields optimal performance, minimizing both the standard deviation and range of fluctuations.

Experimental analysis revealed that optical power ratios critically affect ranging accuracy. We conducted controlled experiments with reference-to-signal power ratios of 50:50, 40:60, 30:70, 20:80, 10:90, and 5:95 while maintaining other parameters. The experimental results are shown in Figure 7. The results indicate that when the power ratio is close to 1:1, the standard deviation and range of the ranging results are close to the optimal values. This is consistent with the result in Section 2.1.2 of the theoretical derivation part mentioned above.

After comprehensively considering the equipment performance and measurement accuracy, we determined that the sampling duration for a single experiment is 4 μs, the sampling rate is 40 GS/s, and the power ratio is 50:50. These systematic experimental results not only fully verify the correctness of the theoretical model but also provide important experimental basis for subsequent optimization of system parameters.

**Figure 8:**
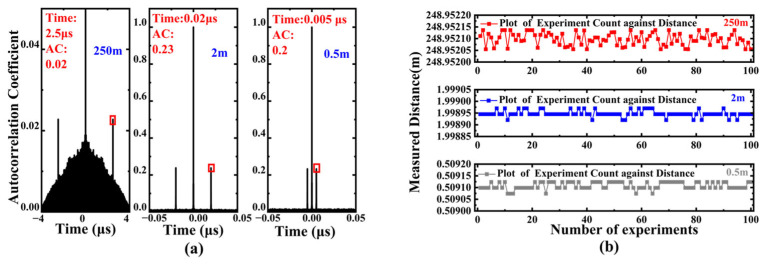
(**a**) Autocorrelation spectrograms at different distances (250 m, 2 m, 0.5 m); (**b**) Stability analysis of 100 repeated measurements under different conditions. The ranging standard deviations at 0.5 m, 2 m, and 250 m (with a signal overlap ratio of 37.5%) were 14.97 μm, 13.05 μm, and 114.28 μm, respectively. By increasing the single measurement duration for the 250 m case to 40 μs (improving the overlap ratio to 93.75%), the standard deviation was significantly reduced to 28.64 μm, demonstrating the consistent and stable measurement performance of this method across different distances.

With the key parameters stabilized at their optimal levels, we evaluated the system performance at multiple distances, including 0.5 m and 2 m. During data processing, according to the formula $\delta L=\frac{c}{2f_{s}}$, the accuracy of this method depends on the sampling rate. While the fundamental temporal resolution of our system remains constrained by the hardware sampling rate ($f_{s}$ = 40 GS/s), we employed cubic spline interpolation combined with a peak fitting algorithm to refine the estimation of the correlation peak position within the sampling intervals. This processing method improves the accuracy of delay estimation. It should be emphasized that this represents an improvement in measurement precision through sophisticated signal processing, rather than an enhancement of the system’s inherent temporal resolution. The experimental results shown in Figure 8a indicate that this method can achieve stable and effective measurements, thereby verifying the feasibility of this measurement method.

To minimize random errors in single experiments, 100 repeated experiments were conducted under conditions ensuring optical path stability and consistency. The experimental results presented in Figure 8b confirm the good stability of this measurement method. Furthermore, to verify the system’s effectiveness in long-distance measurements, a single-mode fiber approximately 250 m long was used to replace the delay line for testing, with the measurement repeated 100 times. The standard deviations corresponding to 0.5 m, 2 m, and 250 m were 14.97 µm, 13.05 µm, and 114.28 µm, respectively. For these measurements, the signal duration for a single measurement was consistently 4 µs, ensuring overlap ratios of 99% at 0.5 m and 2 m, while the signal overlap ratio at 250 m was only 37.5%. Consequently, the standard deviation increased significantly. This observation aligns with our simulation findings regarding overlap ratio, confirming that a smaller overlap ratio leads to a poorer signal-to-noise ratio, thereby affecting measurement precision.

To mitigate the impact of low overlap ratio, the single measurement time for the 250 m case was increased to 40 µs, achieving an overlap ratio of 93.75%. After repeating the measurement 100 times, the results shown in Figure 8b indicate that the standard deviation decreased to 28.64 µm. This demonstrates that under consistent conditions, the method can maintain stable measurement performance across different distances. Due to limitations in the laboratory’s computational capabilities, the overlap ratio could not be further increased to 99%. It should be noted that during data processing, to account for the characteristic that the time of flight in practical measurements includes both the emission and return times, the aforementioned 0.5 m, 2 m, and 250 m distances correspond to actual fiber lengths of approximately 1 m, 4 m, and 500 m, respectively. During processing, the delay time obtained through autocorrelation calculation was halved to conform to the actual time-of-flight characteristics. Therefore, the delay times appearing in the autocorrelation spectrogram correspond to 1 m, 4 m, and 500 m.

### 3.2. Optical Probe Experiment

**Figure 9:**
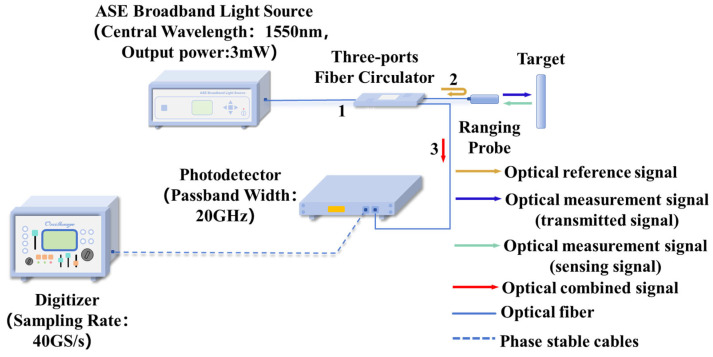
Schematic of the non-contact ranging system. ASE random source emits light through a 3-port circulator to a fiber probe with a Fresnel-reflective end-face. The end-face splits light into a reference beam (reflected internally) and a sensing beam (reflected externally from the target). Both beams combine, return via the circulator, and are converted and digitized for delay estimation.

We replaced the time-delay device with a fiber-optic probe. The configuration of this distance-measurement system is shown in Figure 9. The B-ASE random spontaneous emission light source serves as the signal light source of the system. The optical signal then enters a 3-port circulator. At port 2 of the circulator, the optical signal enters the probe. A Fresnel reflection surface is set at the end-face of the probe. Through this reflection surface, the emitted light source is split into two parts. One part is reflected into port 3 of the circulator after passing through the Fresnel reflection area on the end-face, forming the reference signal. The other part penetrates the Fresnel end-face, irradiates the surface of the object to be measured. Part of the optical signal is reflected from the surface of the object to be measured and re-enters the end-face of the probe, forming the sensing signal. The reference signal and the sensing signal are combined at the end-face of the probe and then enter the photoelectric converter through port 3 of the circulator. The converted electrical signal is connected to a digitizer.

## 4. Results and Discussion

**Figure 10:**
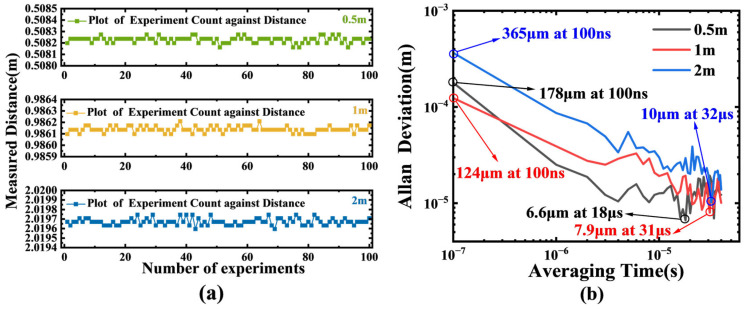
Probe system performance and stability analysis: (**a**) 100 repeated measurement results at 0.5 m, 1 m, and 2 m. The standard deviations of 22.11 µm, 22.25 µm, and 32.36 µm, respectively, confirm the micron-level accuracy of the probe system at various distances. (**b**) Allan deviation analysis. The plot shows that the measurement stability improves with longer averaging time, with the Allan deviation dropping below 10 µm at average times of ~30 µs, characterizing the system’s long-term precision and noise floor.

Different from the verification experiment with the time-delay line, the signal light emitted by the probe can be affected by various environmental factors, which may lead to measurement failures. Therefore, based on the probe-based measurement system and the existing laboratory conditions, we set measured distances of approximately 0.5 m, 1 m, and 2 m to test the measurement performance of the system at different distances. The measurement duration for a single test was 4 μs, and the measurement was repeated 100 times. Finally, the measurement results for 0.5 m, 1 m, and 2 m are shown in Figure 10a. The standard deviations corresponding to the three groups of distances are 22.11 μm, 22.25 μm, and 32.36 μm, respectively. To conduct a stability analysis of the measurement system, we evaluated the Allan Deviation. The entire measurement consists of 4000 independent data points, with each point being collected over a time of 100 ns, and the total duration is approximately 400 μs. The extracted Allan deviation is shown in Figure 10b. At an average time of 100 ns, the Allan deviations for 0.5 m, 1 m, and 2 m are 178 μm, 124 μm, and 365 μm, respectively. Subsequently, these three values decrease to 6.6 μm, 7.9 μm, and 10 μm at average times of 18 μs, 31 μs, and 32 μs, respectively. At relatively short average times, the Allan deviation decreases as the average time increases.

**Figure 11:**
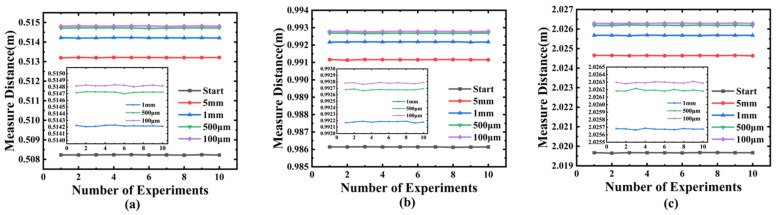
Displacement resolution tests from different initial positions: (**a**–**c**) Fluctuation of measured displacements with 0.5 m, 1 m, and 2 m as initial positions. The system was tested for 5 mm, 1 mm, 500 µm, and 100 µm movements. With a 40 µs single-measurement time, the maximum standard deviation across all tests was 8.17 µm.

Finally, to test the accuracy of this method in practical applications, we used the above-mentioned 0.5 m, 1 m, and 2 m as the initial positions, respectively, and conducted measurements using a precision displacement platform. The displacement amounts were set to 5 mm, 1 mm, 500 μm, and 100 μm. Meanwhile, based on the results of the Allan deviation mentioned above, we set the single-measurement duration to 40 μs and conducted a total of 10 measurements. The experimental results are shown in Figure 11. In the measurement results of the above three groups, the maximum standard deviations of the three groups are 7.65 μm in the 0.5 m range, 6.56 μm in the 1 m range, and 8.17 μm in the 2 m range. Compared with the preliminary measurement at 4 μs, the system accuracy has significantly improved.

Since the standard deviation σ is directly related to the measurement uncertainty $u_{a}$, the 95% confidence interval as an example was used, $u_{a}=2\sigma /\sqrt{Q}$, where Q  represents the number of measurements. The uncertainty of the above measurements is given in Table 3.

**Figure 12:**
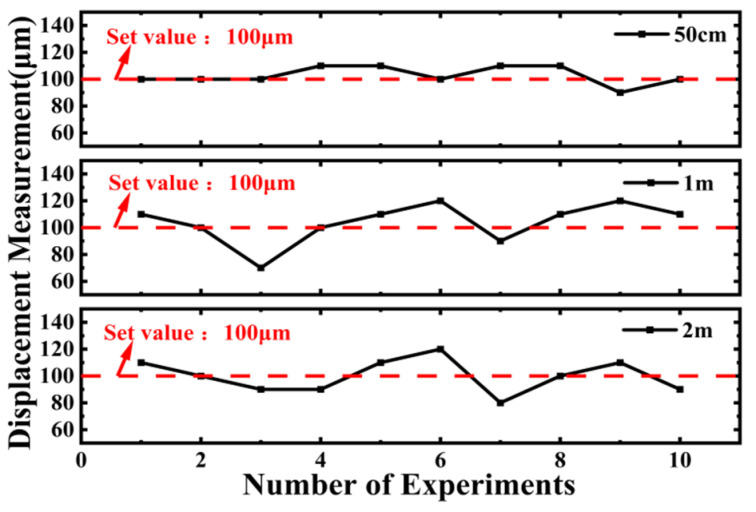
Displacement error for 100 µm movements from different initial positions: The error fluctuation for a 100 µm displacement from 0.5 m, 1 m, and 2 m initial positions shows a maximum error of 30 µm.

At the same time, as can be seen from the error fluctuations in Figure 12 when moving 100 μm from the initial positions of 50 cm, 1 m, and 2 m, the maximum error value is 30 μm and occurs at a measurement distance of 1 m. Therefore, this method of measurement not only has good stability but also the error value does not increase with the increase in distance, providing the possibility for fast precision measurement.

## 5. Conclusions

This paper presents a method that transforms the statistical characteristics of optical random noise into a carrier for precise ranging information, demodulating absolute distance through an autocorrelation algorithm. Through systematic optimization, the optimal parameters were determined as a 50:50 beam-splitting ratio, a 40 GS/s sampling rate, and a 40 μs single-measurement duration. Under these conditions, repeated measurements at 0.5 m, 1 m, and 2 m demonstrated a measurement accuracy of 30 μm with a standard deviation of 8 μm. For ultra-fast measurement scenarios, a reduced acquisition time of 4 μs was also tested, yielding a standard deviation of 32 μm over the same distances. The system exhibits scalable performance: increasing the sampling rate significantly enhances accuracy, while extending the acquisition time effectively expands the measurable range. This flexibility allows a single system to adapt to diverse application requirements, reducing the need for multiple specialized instruments and offering high practical value for industrial applications where cost control and operational adaptability are critical.

The system employs a coaxial optical path design to minimize interference from environmental factors such as temperature variations, thereby enhancing stability and robustness in practical deployment. However, its maximum effective range is primarily constrained by the signal-to-noise ratio. Although the theoretical range can reach the kilometer level, practical performance is affected by optical power attenuation and a reduced signal overlap ratio, which ultimately degrade measurement accuracy.

## Figures and Tables

**Table 1:** This paper compares with the existing technology.

Method	Measured Distance	Standard Deviation	Single Measurement Time
OSCAT [30]	22 mm	4 μm	10 s
FSI [31]	73.51 mm	0.19 μm	1 s
OCMSI [32]	35 m	60 μm	second level
CPSI [33]	18 cm	2.51 μm	1.76 ms
SI [34]	70 m	30 μm	100 ms
This work	2 m	8 μm	40 μs

**Table 2:** Comparison Table of Sampling Rate and Measurement Accuracy Values.

Sampling Rate (GS/s)	Measured Distance (m)	Standard Deviation (µm)	Distance Range (µm)
1.25 GS/s	1.3 m	5268 µm	25442 µm
10 GS/s	1.3 m	255 µm	1344 µm
20 GS/s	1.3 m	80 µm	384 µm
40 GS/s	1.3 m	20 µm	96 µm

**Table 3:** Measurement results at different measurement distances.

Distance	Uncertainty	Standard Deviation	Single Measurement Time
0.5 m	4.84 μm	7.65 μm	40 μs
1 m	4.15 μm	6.56 μm	40 μs
2 m	5.17 μm	8.17 μm	40 μs

## Data Availability

The original contributions presented in this study are included in the article. Further inquiries can be directed to the corresponding authors.
